# Aspheric Surface Measurement Using Capacitive Sensors

**DOI:** 10.3390/s17061355

**Published:** 2017-06-11

**Authors:** Daocheng Yuan, Huiying Zhao, Xin Tao, Shaobo Li, Xueliang Zhu, Chupeng Zhang

**Affiliations:** 1 State Key Laboratory for Manufacturing Systems Engineering, Xi’an Jiaotong University, Xi’an 710049, China; 18608160633@wo.cn (D.Y.); xueliang.zhu@foxmail.com (X.Z.); zcp1988123@126.com (C.Z.); 2 Institute of Machinery Manufacturing Technology, China Academy of Engineering Physics, Mianyang 621000, China; cztaoxin@foxmail.com (X.T.); qhyouxianyuan@163.com (S.L.)

**Keywords:** aspheric surface measurement, electromagnetic surface, capacitive probe, error compensation

## Abstract

This paper proposes a new method for the measurement of spherical coordinates by using capacitive sensors as a non-contact probe solution of measurement of aspheric surfaces. The measurement of the average effect of the capacitive probe and the influence of capacitive probe tilting were studied with respect to an eccentric spherical surface. Based on the tested characteristic curve of the average effect of the sphere and probe, it was found that nonlinear and linear compensation resulted in high measurement accuracy. The capacitance probe was found to be trying to fulfill a need for performing nm-level precision measurement of aspheric electromagnetic surfaces.

## 1. Introduction

Aspherics are optical components whose surfaces deviate from those of spherics [1]. Compared to spherical optics, they are more flexible, have a larger degree of freedom, and exhibit more diversity. Aspherics are used in the optical field to correct aberrations effectively and reduce the number of optical components in optical systems [2,3]. The development of optical precision fabrication has enabled the common use of aspheric lenses and surfaces in modern high-quality optics. With the wide application of aspheric lenses, high precision aspheric surface shape error has been identified as a key factor affecting the quality of work. Therefore, aspheric surface testing techniques have become a significant area of research in the field of optical engineering [4].

Several existing methods can be used to measure aspheric surfaces. Interferometers can accurately measure plano, spherical, and small departure aspheric surfaces [5]. However, null correction is normally required for accurate interferometric measurements of large departure aspheres [6]. This method typically requires a specially designed and customized compensator, computer generated hologram (CGH), or other auxiliary components [7]. Another approach used to measure aspheres is that of profilometry [8]. This method provides simple and accurate scans of the asphere with the possibility of mapping the whole surface. The probes used in this method are divided into two general types: contact and non-contact probes. Contact probes are capable of highly accurate measurements, but risk damaging the surface [9]. For scanning measurement of aspheres without utilization of CGH, [9] proposes a new elliptical setup using a laser interferometer and a set of scanning mirrors, operating diameters from 10 mm to 55 mm with a local uncertainty <50 nm, and the global uncertainty is estimated to be in the range of 100 nm.

It is desirable that such metrology systems be non-contact, highly accurate, and possess fast measurement speed [10,11,12]. For the measurement of aspheric and free-form optics and molds, reference [12] introduces a new optical technique called confocal tracking, consisting on tracking the focus on the sample. Confocal tracking provides shape measurements with nm-level accuracy and acquisition speeds of 1 mm/s typically, but it is large, complex, and a proprietary technology. Achievable accuracy is determined by the type of probe used and the reference datum of the measurement system. The non-contact probes play a decisive role. An optical probe is the main choice for high accuracy measurement of electromagnetic surfaces [11]. However, a change in the skew of the measured surface cannot be avoided. As stated in [10], a tilt dependency of approximately 550 nm exists at 5°, which may be compensated to an uncertainty of 35 nm. Nanometer Accuracy Non-contact Measurement of Freeform Optical Surfaces (NANOMEFOS) (developed by TNO (Delft, The Netherlands) in conjunction with Zeeko) has objective accuracy 30 nm (2σ). However, the complete measuring machine has an uncertainty of 10 nm (2σ) when the surface is perpendicular to the probe, and 35 nm when the surface is at 5°. Of course, NANOMEFOS is extremely bulky, heavy, and expensive, at the same time as you can see, the optical probe is a very important error source. As traditional non-contact probes, capacitive probes have many advantages such as non-contact, high accuracy, short responding delay, and good dynamic adaptability [13]. Their resolution is at the sub-nanometer level; hence, they can easily achieve measurements on a nanometer scale. This has led us to the solution of using capacitive sensors as a non-contact probe. In this paper, a spherical coordinate measurement method is proposed for asphere surface measurements using capacitive probes. Problems relating to capacitive probes are discussed, including average effect and the influence of tilting. By comparing the measurement results from a roundness measuring instrument and capacitive probe, validity of the error compensation method were verified.

## 2. Problems of Aspheric Surface Measurement Using Capacitive Sensors

Capacitance probes have outstanding advantages, but several open questions exist regarding their use in aspheric measurement, which are listed below.

1. The small range of the problem: While a small range is a prerequisite in realizing the measurement process, surface shape measurements need a wide-ranging sensor.
2. The insulating material measurement problem: Capacitance probes apply measurements against electrically conductive materials. When insulating materials are measured, additional processing is necessary. The same thing happens with a scanning electron microscope (SEM). Conventionally non-conductive samples are observed using SEM by simply coating their surface with a conductive film of thickness of 10 nm or less; this article does not explore this problem.
3. The problem of measuring direction tilting: It is difficult to avoid this for curved surface measurement.
4. The convex and concave measurement problem: Although the measurement range is more sensitive for concave measurement, this paper analyzes only the example of convex measurement in terms of the influence of precise similarity measurement and the limitations of the research methods.
5. The average effect: When measuring curved surfaces, there are significant measured value deviations when compared to a flat surface target due to a larger measuring spot of the capacitive probe, called the average effect. Experience shows that this is an important factor influencing measurement accuracy [14]; and it is also the main content of the experimental research in this paper.

### 2.1. Aspheric Surface Measurement Using a Spherical Coordinate Machine with Small Range Capacitive Probes

**Figure 1:**
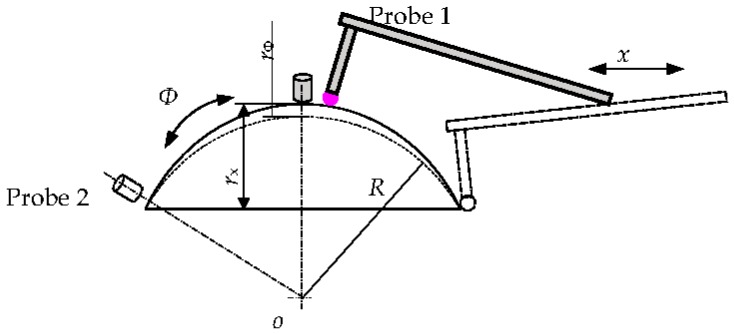
Different ways of using the probe between spherical coordinates and orthogonal coordinates.

The selection and use of a probe is limited by the measurement method. As shown in Figure 1, the profilometry of an aspheric surface (rectangular Cartesian coordinate system) needs a probe (Probe 1) with a wide range (*r*_x_) and high accuracy. This is a difficult choice as it can give rise to errors owing to problems such as wide-ranging normal (vector) changes. However, the spherical coordinates method (with different scanning movement *Ф*) does not require a large ranging probe (Probe 2, *r*_Ф_ << *r*_x_) and acts as the foundation of aspherical measurement using capacitance sensors.

**Figure 2:**
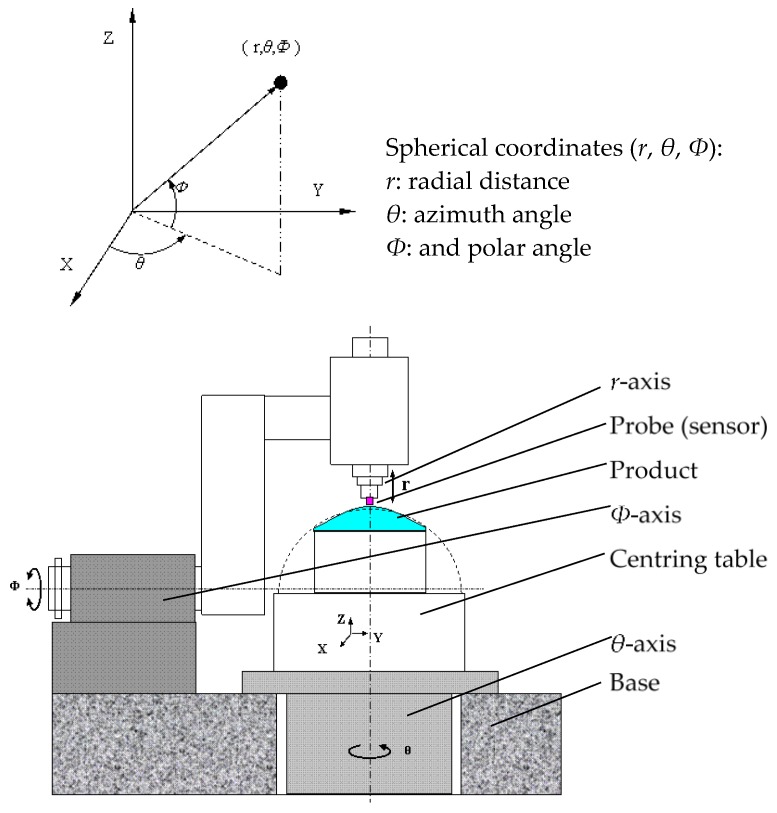
Schematic of spherical coordinate machine used for asphere surface measurement.

Capacitive probes have many advantages such as non-contact, high accuracy, short responding delay, and good dynamic adaptability; however, they have a small measuring range. Since an aspheric surface is a spherical surface whose surface profile has been slightly modified to compensate for any optical aberration created in the spherical lens [15], a spherical coordinate measuring machine setup was proposed (Figure 2).

During measurement, the center of best-fit sphere of the asphere to be measured is set at the center of the spherical coordinates. Capacitive probes can measure the radial distance with an accuracy in the nanometer range when the asphericity is less than the measuring range. Polar axis (*r*-axis) movement and high accuracy measuring can also help avoid this problem, but loss of accuracy is possible. To realize the measurement of aspheric electromagnetic surfaces with nanometer level precision, the rotation precision of the *θ*-axis and *Φ*-axis is required at the nanometer level. Technological advances in manufacturing have already provided significant support, including the air-bearing spindle of 9 nm PV in the axial direction and 12 nm PV in the radial direction [10]. In this paper, the average effect and the influence of tilling are discussed as the main influences on accuracy.

### 2.2. Influence of Measuring Direction Tilting

Like the measurement of flat surfaces, there is a measurement error caused by tilting when measuring curved surfaces. As the measured surfaces are aspheres, the angle between the normal direction of the measured spot and the direction of the capacitive probe is not constant. The influence of the capacitive probe tilting should be compensated to achieve ultra-precision measurement. Consequently, it is important to analyze methods to compensate for the influence of tilting the capacitive probe.

**Figure 3:**
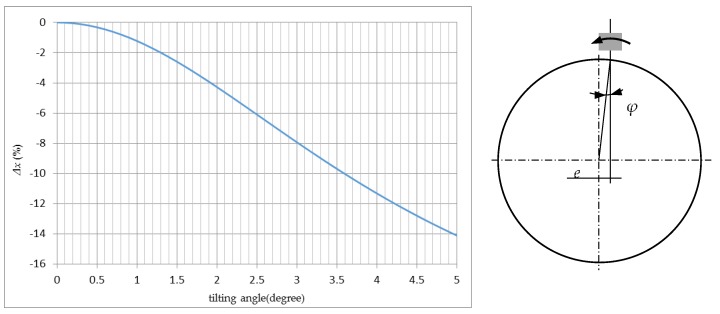
Measurement errors (signal change) with tilting angle.

The principle of capacitive displacement sensor is based on an ideal plate-type capacitor. The two plate electrodes are represented by the sensor and the opposing measurement object. If a constant alternating current flows through the sensor capacitor, the amplitude of the alternating voltage on the sensor is proportional to the distance between the capacitor electrodes. In the case of capacitive sensor tilting, a measurement error must be assumed to reflect the changes in the geometric conditions of the field for the target. The average distance of the sensor remains constant; however, the edge areas move closer or further away from the target [15], which results in field distortions. The signal change (%) can be expressed as follows.

(1) $$\Delta x=100\times (\frac{d}{d_{\max}})\times \left[\frac{1}{1+\frac{r^{2}\tan^{2}\varphi }{4d^{2}}}-1\right],$$

where *Δx* is the signal change; *Φ* is the tilt angle; *r* is the measuring area radius; *d* is the working distance (the real distance instead of the measured value); and *d*_max_ is the sensor measuring range. For *d* = 50 μm, *d*_max_ = 200 μm, *r* = 1300 μm; Figure 3 shows the variation in calculation results for different values of the tilting angle.

Based on the results of Figure 3, an error of 14.1% is caused by an obliquity of 5° corresponding to the corner of the aperture of the aspheric lens, which is an unacceptable error of measurement. For spherical coordinate measurement of asphere surfaces, the change of angle is influenced mainly by the eccentricity. For an eccentricity of 40 μm and sphere *Ф* = 20 mm, the angle change is arcsin (0.04/10) = 0.23°, and the error is only 0.08%. Most of the time, this error can be ignored. The advantages of a small tilting measurement in spherical coordinates can be observed from the subsequent experiment study.

### 2.3. Average Effect

**Figure 4:**
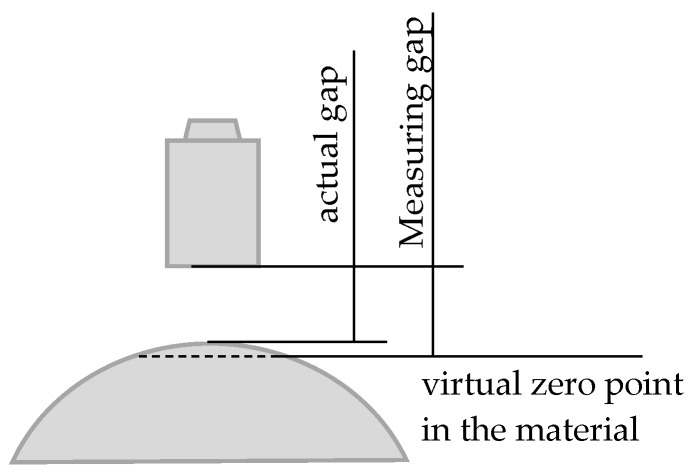
Average effect [14].

When measuring curved surfaces, for example, a sphere surface as opposed to a flat target, there are significant measured value deviations depending on the bending radius. This is caused by various effects, such as the concentration of the field lines at the highest point, or a capacitance increase owing to a larger measuring spot. In reality, it can be assumed that the bending radius results in a virtual zero point—i.e., the sensor value 0—can no longer be achieved. Due to the integrating function of the capacitive sensor over the measurement surface, the virtual, average measuring plane lies behind the surface line as shown in Figure 4.

For the case of a 200 μm sensor and a sphere with external diameter of 20 mm and gap clearance of 20 μm, an excess of approximately 15% is returned, i.e., approximately 50 μm. This effect can be calculated as per [16], and corresponding characteristics can be calibrated in the measurement. Consequently, when measuring aspheres or spheres using a capacitive probe, the measurement values differ from the real clearance [14]. In this paper, the effect of average was summarized as average effect, as shown in Figure 4. This average effect has a significant impact on high accuracy measurement of aspheric electromagnetic surfaces using capacitive sensors.

**Figure 5:**
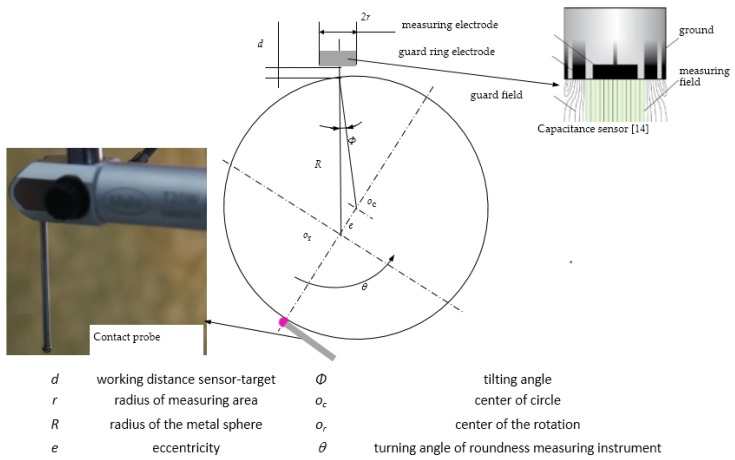
Eccentric sphere synchronization measurement.

To investigate this question, an eccentric spherical surface was employed instead of an asphere surface, as shown in Figure 5. For eccentric measurement, when a sphere rotates, both the measuring distance *d* and tilt angle *Ф* vary, in a manner similar to that of the condition of measuring aspheric surfaces. The measuring distance *d* and tilt angle *Ф* depend on the eccentric value *e* and the rotation angle *θ*, maximum *d* ≥ 2*e*, *Ф* = 0~arc sin (*e*/*R*). So range of *e* are limited by measuring path (maximum *d*) of the sensor. By comparing results from simultaneous measurements performed using a capacitive probe and roundness measuring instrument, the feasibility of using capacitive probes to test the aspheric surface, and the compensation method to address measurement error caused by the average effect was discussed.

There is another average effect different from the above-mentioned phenomenon. This is caused by geometric effects, and is called the geometric average effect because of the geometric average of the measuring area. The calculated error is less than 1 nm under the condition described in this article.

## 3. Experimental Methodology

**Figure 6:**
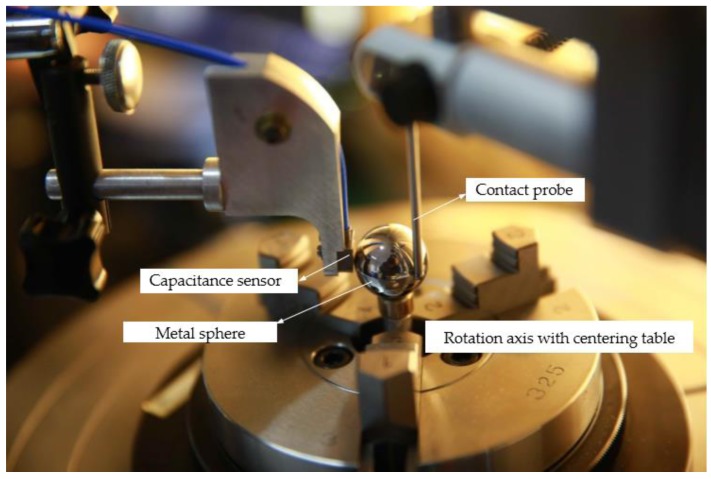
Simultaneous measurement instrument.

A smooth metal sphere (diameter 20 mm) was employed as the measured target. A capacitance displacement sensor Micro-Epsilon (CSH02FL-CRm1,4) and a roundness measuring instrument Mahr MMQ400 were employed as the experimental apparatus shown in Figure 6. The contact probe of MMQ400 was T20w. All experiments were conducted in a temperature-controlled environment (20 ± 0.2 °C) and class 10,000 clean room. The speed of the rotation axis of the MMQ400 was 0.5°/s, the sampling frequency of both probe and sensor were 5 Hz, and sampling quantity was 2–5 × 360°. Detailed parameters of both the probe and sensor are shown in Table 1.

The experimental apparatus is shown in Figure 6.

During the experiment, the measured sphere was attached to the rotating platform, and the equator of the sphere was chosen as the measured surface. The axis of the capacitive sensor, the core of the metal sphere, and the probe center of roundness measuring instrument were set on the same plane using the visual images. When the smooth sphere rotated, both the capacitive probe and contact probe of the roundness measuring instrument simultaneously measured the equator of the sphere. The rotation angle and profile were obtained from the system of the roundness measuring instrument. The data of the capacitive probe were obtained, and the value of the measured spherical surface was expressed in the polar coordinate system. For simultaneous measurement, both the sampling frequencies were set to the same value. The measured results of the roundness measuring instrument were considered standard, and the accuracy of the capacitive probe was estimated with respect to this. For comparability, two types of tests—centric and eccentric—were implemented.

### 3.1. Correction of Average Effect 

**Figure 7:**
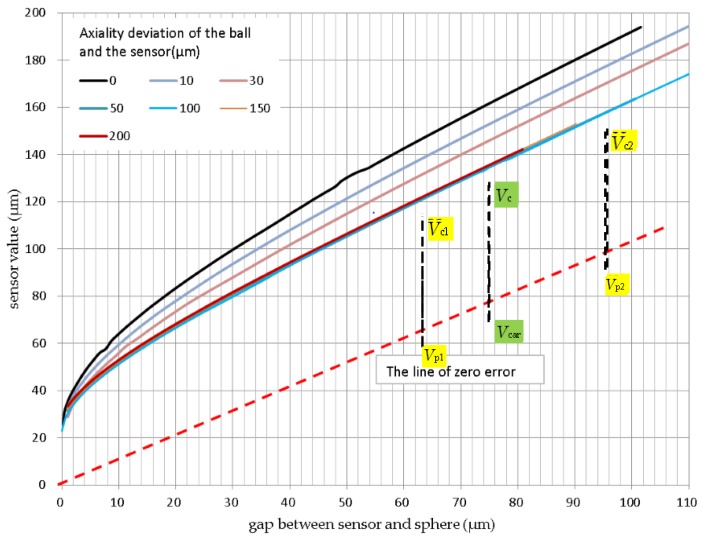
Error characteristic of a capacitive sensor on sphere (average effect).

The error characteristic of a capacitive sensor on sphere was the basis of error correction. Hence, a separate characteristic test using a 20 mm diameter metal sphere and CSH02FL (MICRO-EPSIL, Ortenburg, Germany) sensor was implemented. Figure 7 shows the results of the test. The measured results suggest that a large error existed between the actual and ideal characteristics, and this changed when a different sensor was setup on the same sphere. For spherical (aspherical) surface measurement, it was necessary to implement error compensation, which is comprised of a specific error compensation for every specific measurement, including workpieces, the sensor and its setup, and in situ compensation. Absolute measurement features of the capacitance sensor also requires error compensation.

A specific error compensation method is associated with the workings of a sensor, such as the use of a measuring range of 50 μm or 5 μm, working distance (gap) of 10 μm or 60 μm, etc. In general, a large working distance and small scope of working help achieve higher compensation accuracy. It is important that the error characteristics relate to the installation of the sensor relative to the workpiece. Therefore, it was necessary to realize the in situ error compensation in the measurement.

In view of the eccentric sphere synchronization measurement, two kinds of error compensation methods were used: a nonlinear compensation of wide range, and small-scale linear compensation to validate the error compensation.

#### 3.1.1. Nonlinear Compensation

The correction coefficient test of the average effect was with respect to the 20 mm diameter metal sphere and capacitance displacement sensor of Micro-Epsilon (CSH02FL-CRm1,4) (MICRO-EPSIL, Ortenburg, Germany). The correction factor *f* is given by

(2) $$f=v_{p}/v_{cc},$$

(3) $$v_{ca}=fv_{c},$$

where *v_p_* is the gap clearance value obtained from MMQ400 Probe/T20w (Mahr GmbH, gottingen, Germany) as the true value of the capacitance sensor absolute measurement; *v_cc_* is the capacitance sensor value under *v_p_* during the calibration; and *v_ca_* is the actual displacement value of the capacitance sensor after error compensation. For a known *f*, we can obtain the actual displacement *v_ca_* from the capacitance sensor measuring value *v_c_* during the measurement.

**Figure 8:**
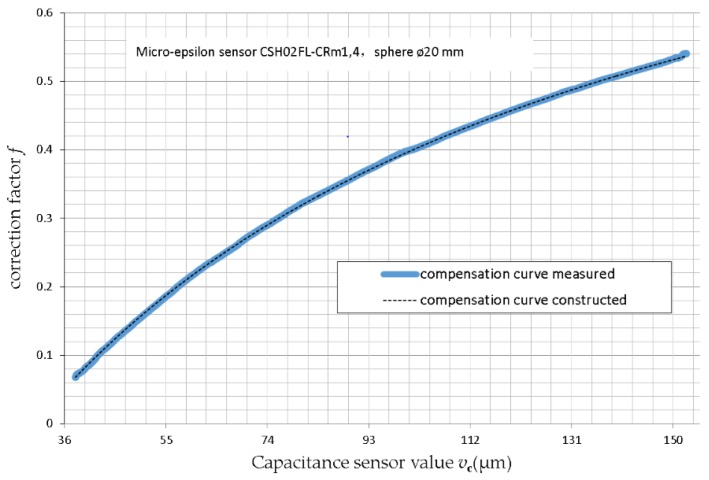
Nonlinear error compensation coefficient of a capacitive sensor on sphere (average effect).

Figure 8 shows the compensation curve obtained from the eccentric sphere synchronization measurement using Equation (2), for a measuring range of capacitance sensor *v_c_* from 38 µm to 152 µm (not corrected, eccentricity 41 μm), and gap clearance value *v_p_* obtained from MMQ400 Probe/T20w as the true value of the capacitance sensor absolute measurement. The influence of the tilt was very small, and the data processing was ignored.

The compensation curve was constructed using empirical Equation (4) based on the measured compensation curve. This was done to make the error compensation process easier, but at the expense of a small additional error. Of course, a more accurate compensation curve could be constructed, but is not dealt with in-depth in this study.

(4) $$f=1-0.4031\times (0.945+\frac{200-v_{c}}{80.4+v_{c}})$$

Using the compensation curve constructed using Equation (4), every *v_c_* could be corrected easily.

#### 3.1.2. Linear Compensation

As shown in Figure 7, when the capacitance sensor operates in a small measuring range, the error characteristic is assumed as linear. Two calibration measurement points are selected, say (*v_c_*_1_, *v_p_*_1_) and (*v_c_*_2_, *v_p_*_2_), and the following parameters are computed.

(5) $$k=\frac{v_{p2}-v_{p1}}{v_{c2}-v_{c1}}$$

(6) $$v_{car}=v_{p1}+k(v_{c}-v_{c1})$$

According to compensation Equations (5) and (6), every *v_c_* could be corrected easily, and *v_c__ar_* was the actual measured value of the capacitance sensor after linear error compensation. For the same measurement range, the linear correction accuracy could be improved by increasing the number of calibration measurement points from two to three, or more. In the case of linear compensation, the actual zero position of the capacitance sensor was not considered, and the shape measurement was not impacted. In the case of measurement using the spherical coordinate machine shown in Figure 2, the two calibration measurement points (*v_c_*_1_, *v_p_*_1_) and (*v_c_*_2_, *v_p_*_2_) could be captured from the sensor and *r* axis.

### 3.2. Centric Measurement

**Figure 9:**
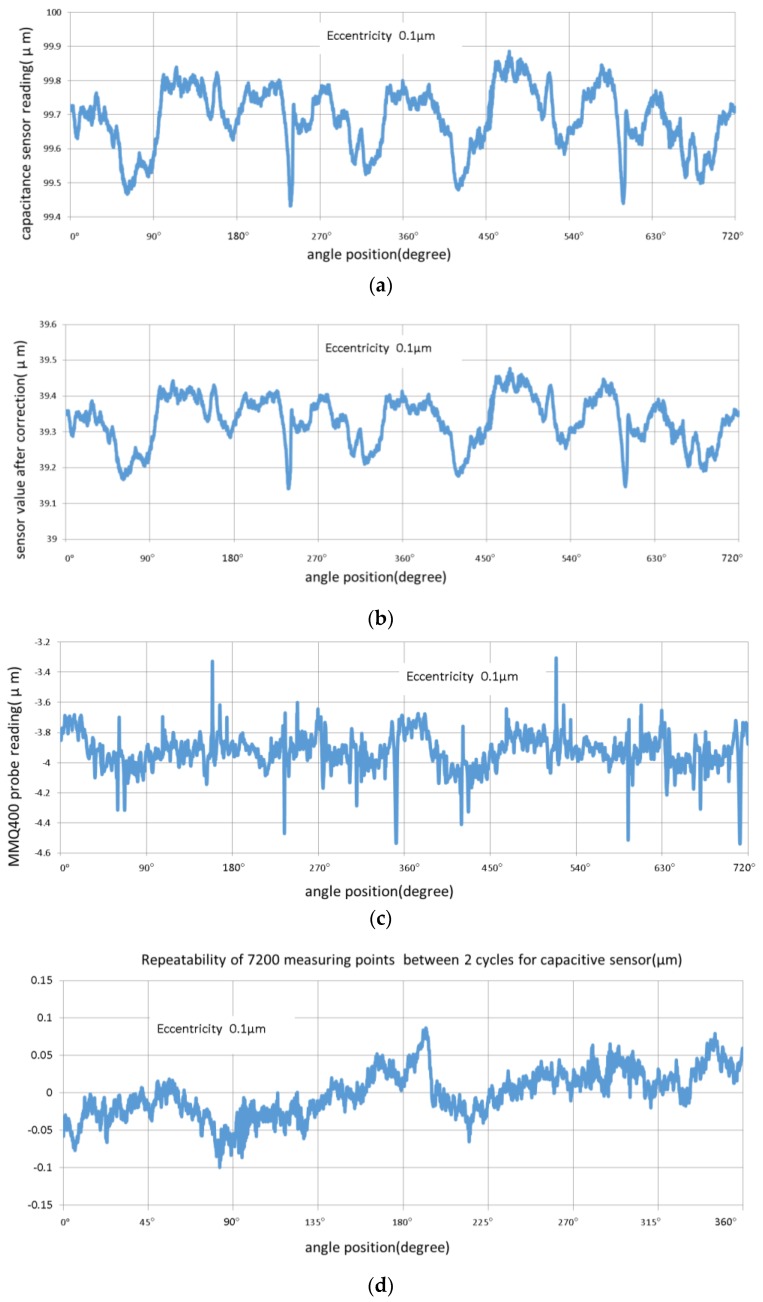

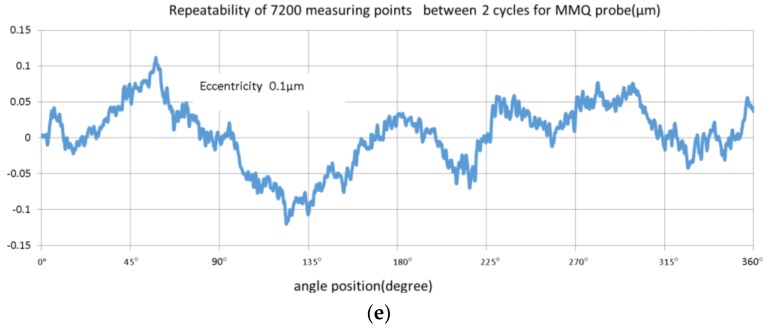
Centric measurement with MMQ400 Probe/T20w and capacitive sensor: (**a**) Capacitance sensor readings in two cycles; (**b**) Capacitance sensor value after nonlinear correction in two cycles; (**c**) MMQ400 probe reading in two cycles; (**d**) Repeatability of 7200 measuring points between two cycles for capacitive sensor after nonlinear correction; and (**e**) Repeatability of 7200 measuring points between two cycles for MMQ probe.

Centric measurement with no changes of eccentricity and tilt can reflect the quantitative characteristics of two kinds of probe, and the uncertainty level of probes is shown in Figure 9.

Every point of centric measurement in every cycle from one of the two probes is sampled under the repeatability condition on similar objects for each of the angles. Repeatability of probes is addressed with two cycles of 7200 points, the difference between the two cycles is addressed to eliminate the influence of shape errors, and repeatability is calculated by max-min not max(absolute values) from d and e. The evaluation of the repeatability like this is for focusing on characteristics of the two probes. If a repeatability of shape measurement is required, more sweep of measurement is needed.

Repeatability of the test for the MMQ probe was approximately 0.24 μm (a sample with a smaller cycle drift was selected as the result, and MMQ probe drift caused a repeatability error of 0.33 μm or larger), which exceeded 0.15 μm, the specification of the MMQ400 Probe/T20w. Based on the square root of the sum of squares method, 0.24^2^ − 0.15^2^ = 0.19^2^, the measurement was associated with an uncertainty of at least 0.19 μm. Repeatability for the capacitive sensor was approximately 0.18 μm, which implied that the sensor was good after removing the 0.19 μm uncertainty. It was less than 0.01 μm as per the specifications of the Micro-Epsilon capacitive sensor. At the same time, spherical measurement exhibited ideal shape measuring precision using a capacitive sensor under centric measurement.

### 3.3. Eccentric Measurement

The eccentric measurement compared the two kinds of probe measuring instruments with the actual characteristic of the eccentric spherical (aspherical), and we discuss the capacitance probe measurement uncertainty characteristic of an aspheric surface. With respect to the capacitance probe average effect, two different error compensation methods and comparison based on application and error characteristics are discussed. For these purposes, and to simplify the process of experimental comparison, a steel ball with a diameter of 20 mm, and eccentric measurement choice of eccentricity 12 μm and 37 μm were used.

**Figure 10:**
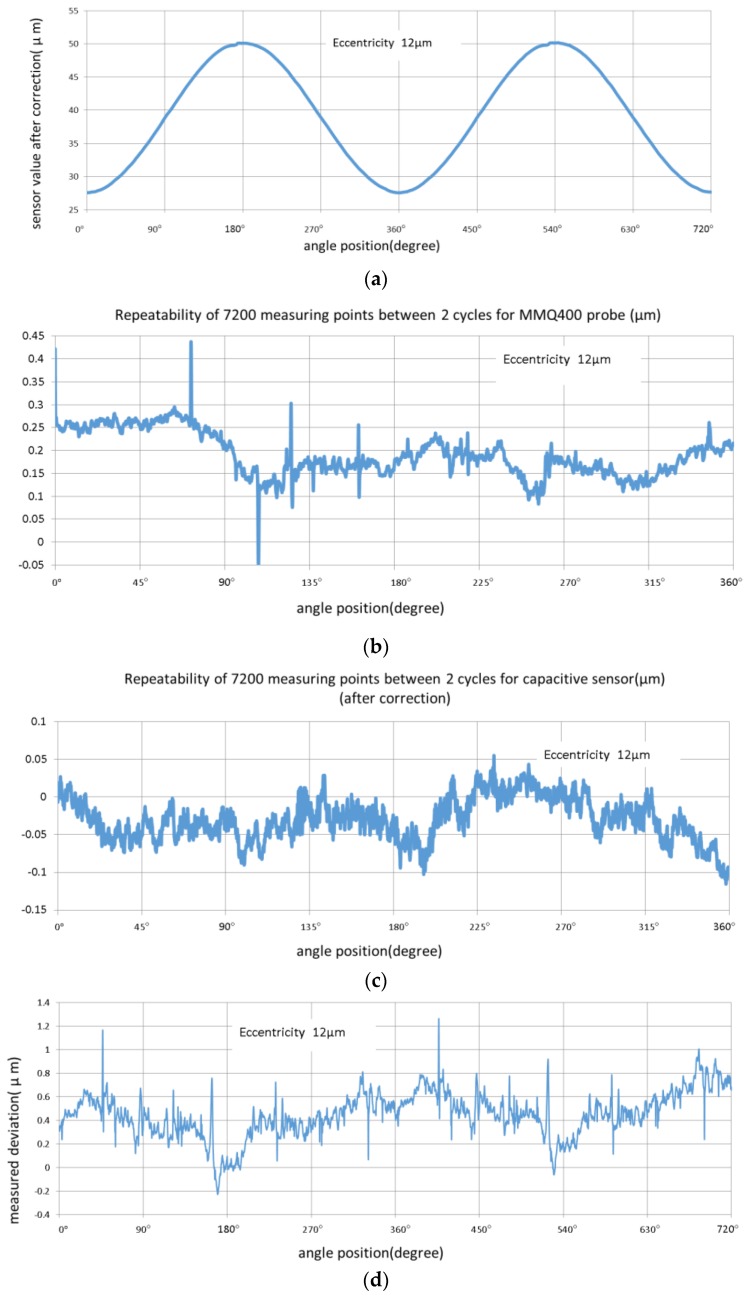
Eccentricity 12 μm measurement with MMQ probe and capacitive sensor: (**a**) Capacitance sensor values after nonlinear correction in two cycles; (**b**) Repeatability of 7200 measuring points between two cycles for MMQ probe; (**c**) Repeatability of 7200 measuring points between two cycles for capacitance sensor; and (**d**) Measured deviations between capacitance sensor and MMQ400 probe.

A comparison of the two 12 μm eccentric spherical measurements shown in Figure 10, showed that no special change was observed in their repeatability. However, the MMQ probe’s high frequency (spatial frequency, the same below) repeatability was worse, which could be attributed to the sampling synchronization error. The result indicated the obvious influence of the high frequency signal, and conformed to the characteristics of the two kinds of measuring probes. When the influence of the high frequency signal was removed, the deviation values were found to be approximately 0.6 μm. Nonlinear compensation effects were observed at the 180° position owing to the deviation of the building coefficient curve, and when this factor was removed, the deviation was approximately 0.4 μm. When considering the MMQ probe measurement repeatability, the form measurement results of the two kinds of head agreed. Therefore, there was no obvious eccentric spherical shape error using the capacitance sensor.

**Figure 11:**
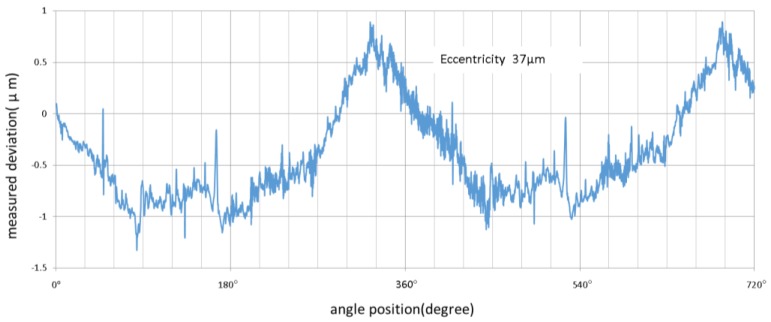
Eccentricity 37 μm measurement deviations between capacitance sensor and MMQ400 probe with capacitive sensor nonlinear correction.

From Figure 11, it was observed that for the measurement and comparison of the 37 μm eccentricity, the shape deviation was more than 2 μm. There was a large deviation between the high-end and low-end of the measuring range. This shows the lack of a wide measuring range with nonlinear compensation for the capacitive sensor.

**Figure 12:**
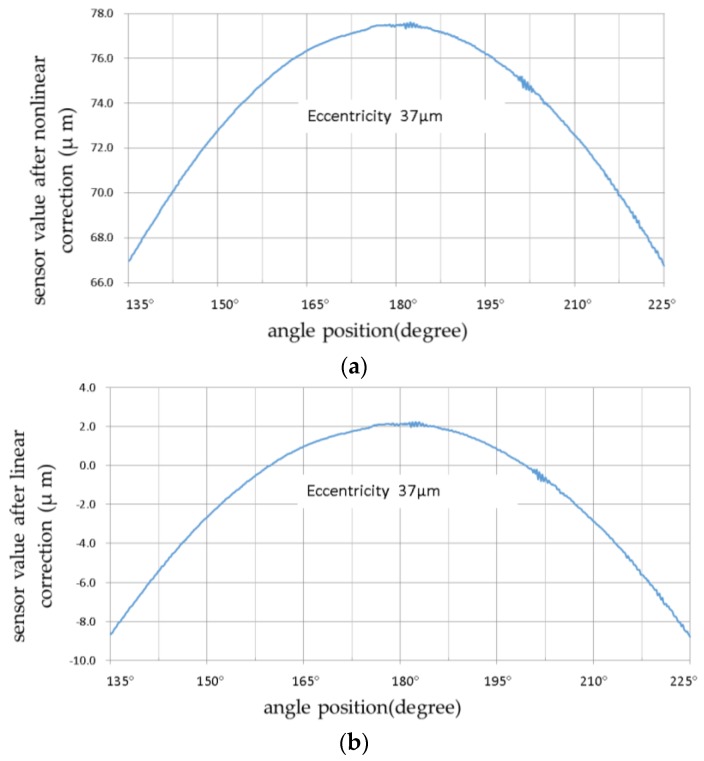

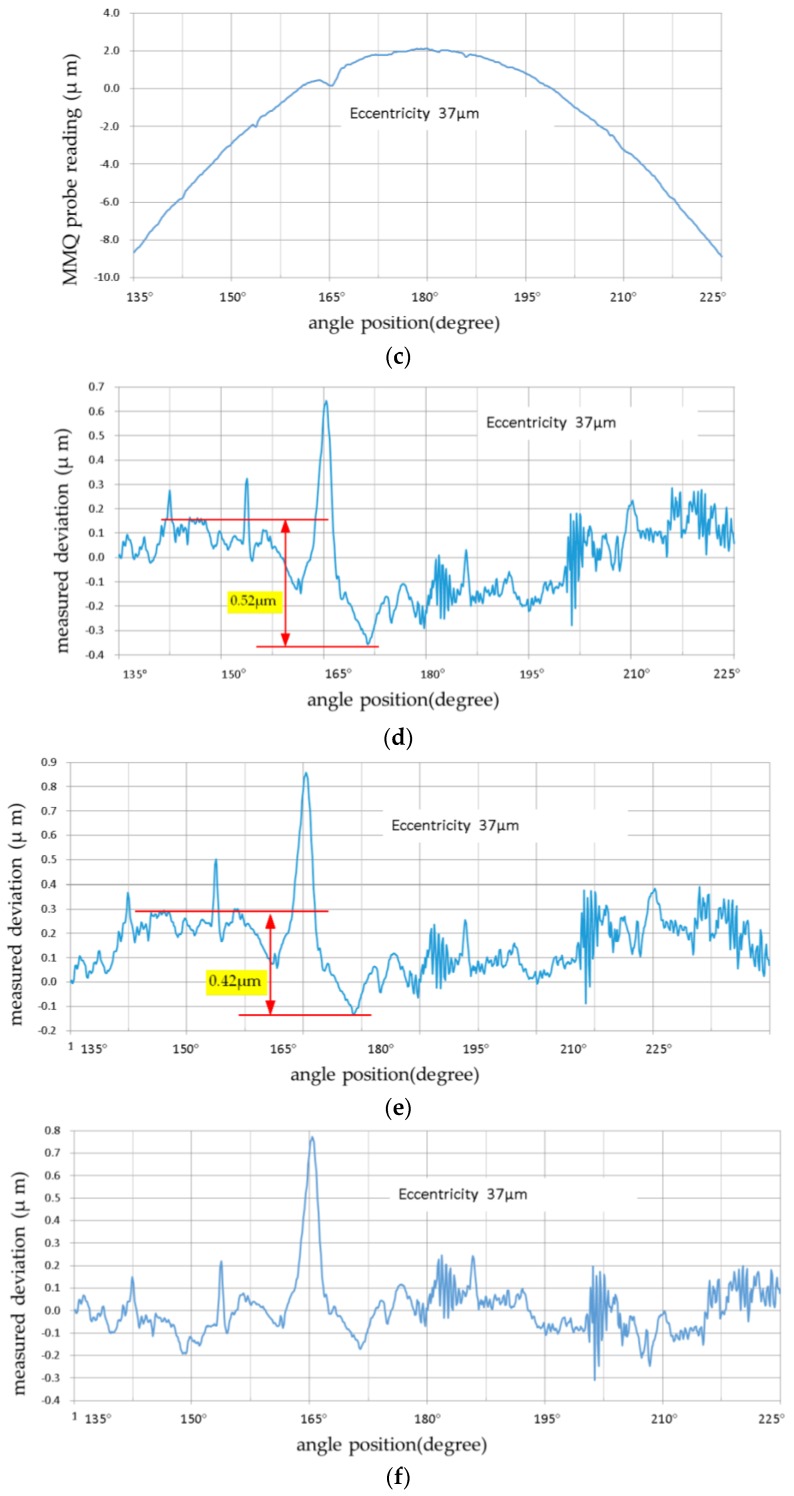
Eccentricity 37 μm measurement results with nonlinear and linear correction: (**a**) Capacitance sensor values after nonlinear correction; (**b**) Capacitance sensor value after linear correction; (**c**) MMQ400 probe readings at eccentricity 37 μm; (**d**) Measurement deviations with capacitive sensor nonlinear correction; (**e**) Measurement deviations with capacitive sensor linear correction (2 points); and (**f**) Measurement deviations with capacitive sensor linear correction (3 points).

Figure 12 shows the results of the application of a small measuring range with linear compensation for the given capacitive sensor for the same 37 μm eccentric measurement when compared with the above-mentioned results.

Figure 12 displays the eccentricity 37 μm measurement results with both nonlinear and linear correction. A comparison of the results showed their different characteristics. In the 10 μm measuring range, both nonlinear correction and linear correction exhibited good compensation accuracy, but linear compensation accuracy was much higher when using three points.

The linear compensation deviation of Figure 12e was much smaller than the nonlinear compensation deviation of Figure 12d. This demonstrates the practical significance of improving the compensation accuracy. The improvement was more obvious at two positions, near 135° and 180°. This suggests that linear compensation performed using three or more calibration measurement points (narrow linear compensation scope) can improve compensation accuracy (Figure 12f). Linear compensation was suitable for small range measurement application of the capacitance probe, and made it easy to obtain higher accuracy compensation. As shown in Figure 7, when the capacitance sensor was working at a distance greater than 30 μm, the measurement error caused by the average effect had an approximately linear relationship with the displacement, and the linearity increased with smaller displacement intervals. This feature provides a convenient way to improve the compensation accuracy of the average effect, and improves the possibility of achieving ultra-precision aspherical measurement applications of capacitance probes.

Removing the influence of the high frequency signal (Figure 12f) decreased the deviation to approximately 0.3 μm, which was close to the MMQ probe repeatability accuracy under the reasonable deviation caused by the sampling difference between the ϕ2.6 active measuring area of capacitance sensor, and the sphere of the contact probe. This implies that the capacitive probe at a small working range could obtain good measuring precision. From observing Figure 12d–f, between 135° and 225° (two measuring positions with the biggest tilting difference) the relative deviation was found to be less than 0.1 μm. This suggests that the influence of the measurement tilting was small and consistent with the analysis results obtained in Section 2.2. Therefore, we conclude that there was no obvious eccentric spherical shape error using the capacitance probe. The two probes showed significant difference in the high frequency signal end. While the capacitance probe was suitable for form measurement, the special capacitance probe was found to be better for roughness measurement [17].

From the above-mentioned results, we conclude that the capacitance probe can be used for eccentric spherical measurements, and good accuracy of shape measurement can be obtained by selecting the appropriate working distance, using a small measuring range, and implementing error compensation of average effect. Thus, from an aspherical shape (low frequency) measurement perspective, capacitance probes may be a good choice for a non-contact precision measuring scheme with an uncertainty close to the measurement repeatability of the capacitance sensors.

## 4. Conclusions

A new method of measurement of aspheric electromagnetic surfaces based spherical coordinate measurement using a capacitive probe was proposed. Taking an eccentric spherical surface as the object of study, the measurement average effect and the influence of the measurement tilting of the capacitive probe were discussed. Centric and eccentric shape measurement precision of a sphere was verified experimentally by comparing the measurement results of simultaneous measurements of the capacitive probe and Mahr MMQ400 roundness measuring instrument.

Aspheric surface measurement using a capacitance probe had a repeatability accuracy of 0.01 μm, and the tilt changes of a small scale had little impact on measurement precision. Aspheric surface measurement using a capacitance probe had a significant average effect error related to surface curvature [14], working distance, and surface setup. This necessitated a probe setup to perform calibration and compensation of the workpiece. Based on the tested characteristic curve of average effect for the sphere and probe, it was found that nonlinear and linear compensation resulted in good measuring accuracy. Therefore, the capacitance probe was found to be trying to fulfill a need of performing nm-level precision measurement of aspheric electromagnetic surfaces.

The use of spherical coordinates measurement to reduce the complexity of the probe application, is advantageous for the tilt control of the capacitance probe, and advantageous to the calibration and compensation of the capacitance probe in the process of measurement. In future studies, constructing a spherical coordinates nano-precision measuring system of aspherical surfaces would be an important challenge.

## Figures and Tables

**Table 1:** Detailed parameters of contact probe and capacitance sensor.

**MMQ400 Probe/T20w**	
Measuring path	±1000 µm
Free travel	±0 to ±1000 µm
Sensitivity deviation	≤0.5%
Measuring force infinitely adjustable	up to 0.12N
Measuring force hysteresis	max 0.006N
Measuring value hysteresis	≤0.03% (max 0.6 µm/2000 µm)
Repeatability	0.15 µm
Linearity deviation	≤0.2% (max 4 µm/2000 µm)
**Micro-Epsilon Capacitive Sensor (CSH02FL-CRm1,4)**	
Measuring range	200 µm
Resolution	0.15 nm (static, 2 Hz)
Temperature stability zero	2.4 nm/°C
Temperature stability sensitivity	–12 ppm/°C
Linearity	±0.05% FSO
Active measuring area	Ø2.6 mm
Guard ring width	Ø1.9 mm
Minimum target diameter	Ø7 mm
